# mRNA-delivered neutralizing antibodies confer protection against SARS-CoV-2 in animal models

**DOI:** 10.1128/jvi.01897-25

**Published:** 2026-01-07

**Authors:** Nicholas C. Hazell, Rachel A. Reyna, Awadalkareem Adam, Srinivasa Reddy Bonam, Jiani Bei, Naveen Kumar, Tina N. Nguyen, Jessica A. Plante, Tong Wu, David H. Walker, Tian Wang, Kenneth S. Plante, Haitao Hu

**Affiliations:** 1 Department of Microbiology and Immunology, University of Texas Medical Branch 547647, Galveston, Texas, USA; 2 Department of Pathology and Experimental Pathology Graduate Program, University of Texas Medical Branch 12338, Galveston, Texas, USA; 3 World Reference Center for Emerging Viruses and Arboviruses (WRCEVA), University of Texas Medical Branch 12338, Galveston, Texas, USA; 4 CSIR - Indian Institute of Chemical Technology (IICT) 62391, Hyderabad, India; 5 R&D Division, Eureka Biotech Inc., Philadelphia, Pennsylvania, USA; 6 Institute for Human Infections and Immunity, University of Texas Medical Branch 551582, Galveston, Texas, USA; University of Michigan Medical School, Ann Arbor, Michigan, USA

**Keywords:** animal models, SARS-CoV-2, antibodies, mRNA

## Abstract

Monoclonal antibodies represent potent biological countermeasures against a wide range of human diseases; however, their clinical application and widespread use are limited by the high cost and complexity of antibody production and manufacturing. The mRNA-lipid nanoparticle (mRNA-LNP) platform offers a versatile strategy for vaccine development and protein-replacement therapies. Since the COVID-19 pandemic, a number of severe acute respiratory syndrome coronavirus 2 (SARS-CoV-2)-neutralizing antibodies have been identified, with several granted emergency use authorization for patients. Here, we report the design and generation of mRNA-LNPs encoding two SARS-CoV-2-neutralizing antibodies, 76E1 and LY1404, which, respectively, target the spike protein’s fusion peptide (FP) and receptor-binding domain (RBD). We demonstrated that a single intramuscular administration of these mRNA-LNPs in mice resulted in robust antibody production that sustained in circulation for 7–14 days. Furthermore, we evaluated protective effects of these mRNA-delivered antibodies in animal models and showed that a single IM dose of mRNA-LNPs encoding LY1404 or 76E1 conferred significant protection against multiple SARS-CoV-2 variants, including Omicron BQ.1 and Delta, in mice and hamsters. Collectively, these findings highlight the potential of mRNA-based antibody delivery for rapid prevention or treatment of pathogenic infections.

**IMPORTANCE:**

Neutralizing antibodies represent potent biological countermeasures against viral infections. However, the high cost of antibody production restricts its clinical accessibility and large-scale application. The mRNA-lipid nanoparticle (mRNA-LNP) offers a versatile platform for developing vaccines and protein-replacement therapies. In this study, we designed and generated mRNA-LNPs encoding two SARS-CoV-2-neutralizing antibodies, 76E1 and LY1404, which target the viral spike protein’s fusion peptide (FP) and receptor-binding domain (RBD), respectively. A single intramuscular administration of mRNA-LNPs encoding LY1404 or 76E1 resulted in rapid antibody production in circulation and conferred protection against multiple strains of SARS-CoV-2 infection in animal models. Our findings highlight the potential of mRNA-based antibody delivery for rapid prevention or treatment of pathogenic infections.

## INTRODUCTION

Since the first monoclonal antibody was approved by the U.S. Food and Drug Administration in 1986 for the treatment of kidney transplant rejection (1), therapeutic antibodies have been extensively developed and manufactured as biological countermeasures against a wide range of human diseases, including pathogenic infections (2). While technologies for monoclonal antibody discovery and engineering have advanced substantially over the past several decades (3, 4), their production and manufacturing remain costly and labor-intensive, thereby limiting the global accessibility of antibody-based therapeutics (5–8).

Severe acute respiratory syndrome coronavirus 2 (SARS-CoV-2), the etiologic agent of COVID-19, has caused a global pandemic with an unprecedented impact on public health worldwide (9–11). To date, a number of SARS-CoV-2-neutralizing antibodies have been identified from convalescent or vaccinated individuals, targeting distinct epitopes on the viral spike (S) protein and exhibiting varying degrees of neutralization breadth (12, 13). LY1404, also known as bebtelovimab, is a potent neutralizing antibody that targets the receptor-binding domain (RBD) within the S1 subunit of the S protein (14). It demonstrated strong neutralizing activity against early SARS-CoV-2 strains and was granted emergency use authorization for treating COVID-19 prior to its discontinuation following the emergence of resistant variants (15, 16). In contrast to the RBD, the S2 subunit of the S protein is more genetically conserved across SARS-CoV-2 lineages (14). Several S2-targeting antibodies have also been identified and isolated from convalescent individuals (17–19), including 76E1, a broadly neutralizing antibody that recognizes the fusion peptide (FP), a small cryptic epitope in the S2 subunit (20, 21).

mRNA-lipid nanoparticles (mRNA-LNPs) represent a versatile platform for developing new prophylactic and therapeutic countermeasures, such as vaccines and protein replacement therapies (21–26). The clinical approval and widespread use of two mRNA-based COVID-19 vaccines marked a major milestone in the successful translation of this technology (27, 28). Beyond vaccines, mRNA-LNPs have also been employed to deliver monoclonal antibodies for combating infectious diseases (29–33). However, it remains unclear whether mRNA-delivered antibodies targeting conserved epitopes of SARS-CoV-2 S protein, such as the FP-targeting antibody 76E1, aside from the RBD-targeting antibodies, can confer rapid protection against SARS-CoV-2 infections. Addressing this question would facilitate the development of antibody-encoding mRNA-LNPs as an alternative or complementary approach to vaccination for achieving rapid protection against pathogenic infections, particularly in high-risk or immunocompromised populations.

In this study, we designed and generated nucleoside-modified mRNA constructs encoding the heavy chain (HC) and light chain (LC) of 76E1 and LY1404 antibodies. These mRNAs were formulated in LNPs for *in vivo* delivery. Our data show that intramuscular (IM) administration of mRNA-LNPs encoding the HC and LC of each antibody results in robust expression of LY1404 or 76E1 in circulation in both mice and hamsters, with minimal or undetectable antibody levels in the respiratory mucosa. We further demonstrate that a single IM dose of mRNA-LNPs encoding LY1404 or 76E1 confers significant protection against multiple SARS-CoV-2 variants, including Delta, Omicron BQ.1, and a mouse-adapted (MA) prototype strain, in hamster and murine models.

## RESULTS

### Design and *in vitro* characterization of mRNAs encoding LY1404 and 76E1 antibodies

**Fig 1:**
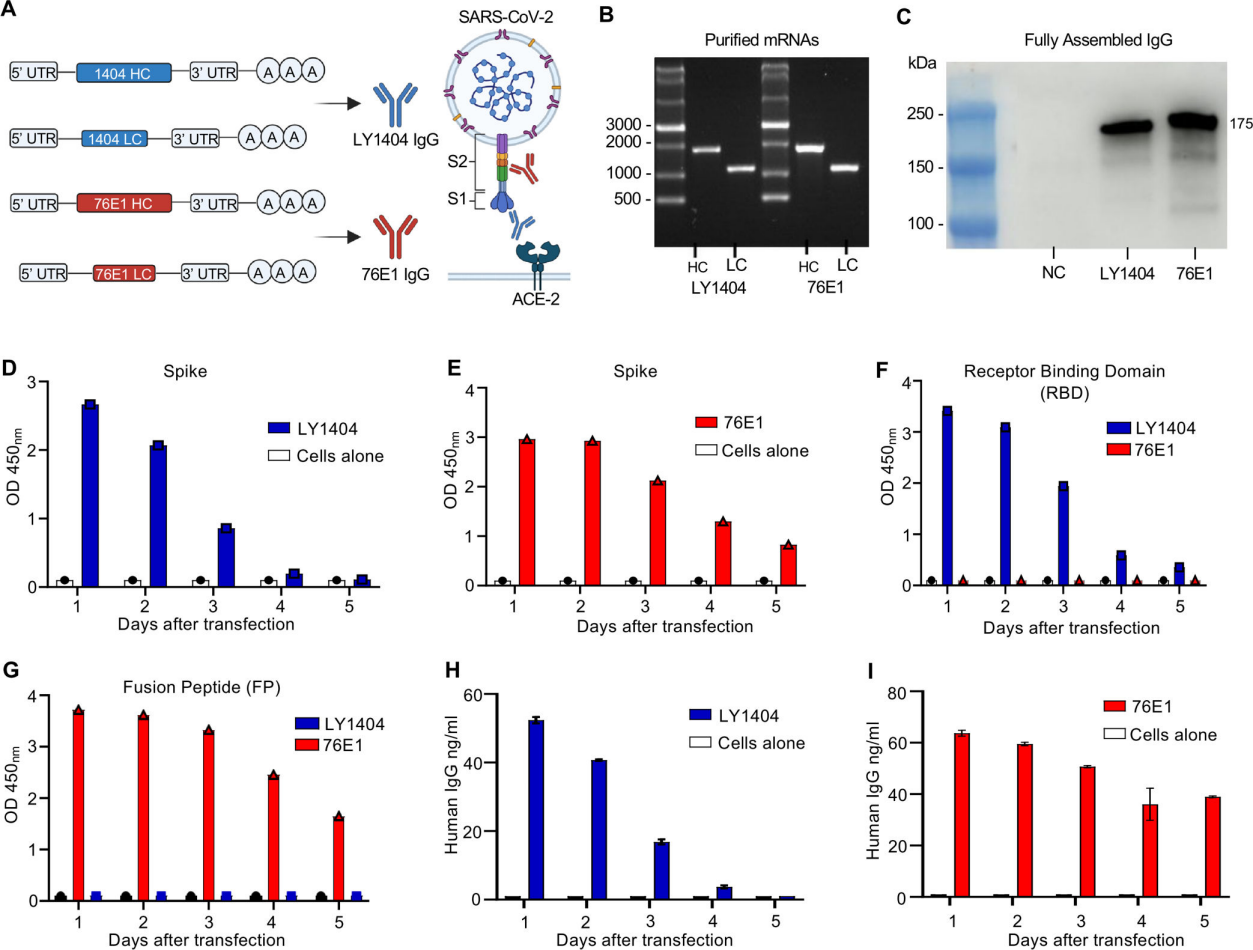
Design and *in vitro* characterization of mRNAs encoding LY1404 and 76E1 antibodies. (**A**) Illustration of mRNA constructs encoding the HC and LC of LY1404 and 76E1 antibodies that respectively target the RBD and FP epitopes of SARS-CoV-2 S protein. (**B**) Gel electrophoresis analysis of purified HC and LC mRNAs. RNA markers were included to determine the sizes of mRNAs. (**C**) Western blot (WB) analysis of antibody production in culture supernatants. 293T cells were transfected with HC and LC mRNA at a 1:1 molar ratio. Cell supernatants were collected 24 h after transfection for WB analysis. Both antibodies were detected by using an anti-human IgG LC antibody. The estimated size of the two fully assembled antibodies is indicated (~175 KDa). NC: negative control (no mRNA). (**D–E**) Kinetics of LY1404 (**D**) or 76E1 (**E**) production in culture supernatants measured by spike-binding enzyme-linked immunosorbent assay (ELISA). 293T cells were transfected with HC and LC mRNA, and antibodies were measured by ELISA at indicated days after transfection. OD450_nm_ was shown for each sample. Cells alone without mRNA transfection were included as a control. (**F–G**) Kinetics of antibody production in culture supernatants measured by RBD (**F**) or FP (**G**) binding ELISA. (**H and I**) Measurement of LY1404 (**H**) or 76E1 (**I**) concentrations in supernatants by human IgG ELISA at indicated days after mRNA transfection. As shown in D–G, ELISA was conducted in singlet. As shown in H–I, quantitative ELISA was conducted with duplicates, and error bars show standard error of the mean. Experiments were performed independently at least twice.

LY1404 and 76E1 are two SARS-CoV-2-neutralizing antibodies that recognize the RBD in S1 and FP in S2 of the S protein, respectively (Fig. 1A). To generate mRNAs expressing these antibodies, codon-optimized HC and LC sequences of LY 1404 or 76E1 were cloned into DNA constructs optimized for mRNA synthesis and translation (34). Each HC or LC sequence included a signal peptide upstream of the variable (VH and VL) and constant (CH and CL) regions, respectively, to promote antibody expression and secretion (Fig. 1A). Linearized DNA templates were used for *in vitro* transcription (IVT), and the resulting mRNAs were precipitated utilizing lithium chloride followed by cellulose-based purification to remove residual dsRNA contaminants (35). Gel electrophoresis and RNA integrity analyses confirmed the high quality and integrity of the purified HC and LC mRNAs (Fig. 1B; Fig. S1).

To assess antibody expression, HC and LC mRNAs of each antibody were co-transfected at a 1:1 molar ratio into 293T cells. WB analysis of culture supernatants harvested 24 h after transfection revealed robust production of fully assembled LY1404 and 76E1 antibodies (Fig. 1C). ELISAs were performed on the same samples to confirm expression kinetics and antigen specificity of the antibodies. First, antigen-binding ELISAs were conducted using plates coated with SARS-CoV-2 S, RBD, or 15-*mer* FP overlapping peptides. The results showed that antibodies produced from LY1404 or 76E1 mRNA-transfected cells both recognized S (Fig. 1D and E; Fig. S2A and B). In contrast, only antibodies produced from LY1404 mRNA-transfected cells bound to the RBD (Fig. 1F; Fig. S2C), whereas only antibodies produced from 76E1 mRNA-transfected cells bound to the FP (Fig. 1G; Fig. S2D), confirming the antigen specificity of each antibody. Second, human IgG ELISA was used to measure the antibody concentrations in culture supernatants. Both antibodies were readily detected at comparable concentrations (55–65 ng/mL) (Fig. 1H and I; Fig. S2E and F). Both antibodies also exhibited similar expression kinetics following mRNA transfection, with levels peaking at day 1 and gradually declining by day 5 (Fig. 1D through I). Collectively, these results demonstrate efficient expression and distinct antigen specificity of LY1404 and 76E1 antibodies produced by cells following mRNA transfection.

### Encapsulation of mRNAs into LNPs for *in vivo* delivery

We next formulated the purified HC and LC mRNAs into LNPs for *in vivo* delivery. The LNP formulation consists of SM102 as the ionizable lipid, DSPC, cholesterol, and DMG-PEG-2K (Fig. S3A). Each HC or LC mRNA was diluted in citrate buffer as the aqueous phase and then rapidly mixed with an ethanol phase containing the four lipids (Fig. S3A). The resulting mRNA-LNPs were prepared and characterized for particle size, polydispersity index (PDI), and RNA encapsulation efficiency (EE%) (Fig. S3B through D). The mRNA EE% was determined using the RiboGreen assay based on an RNA standard curve (Fig. S3C). All four mRNA-LNPs exhibited comparable particle sizes (102–110 nm), PDI (0.107–0.134), and RNA EE% (94%–96%) (Fig. S3B through D). These formulated mRNA-LNPs were used in subsequent *in vivo* studies.

### *In vivo* antibody expression in mice and hamsters following mRNA-LNP administration

**Fig 2:**
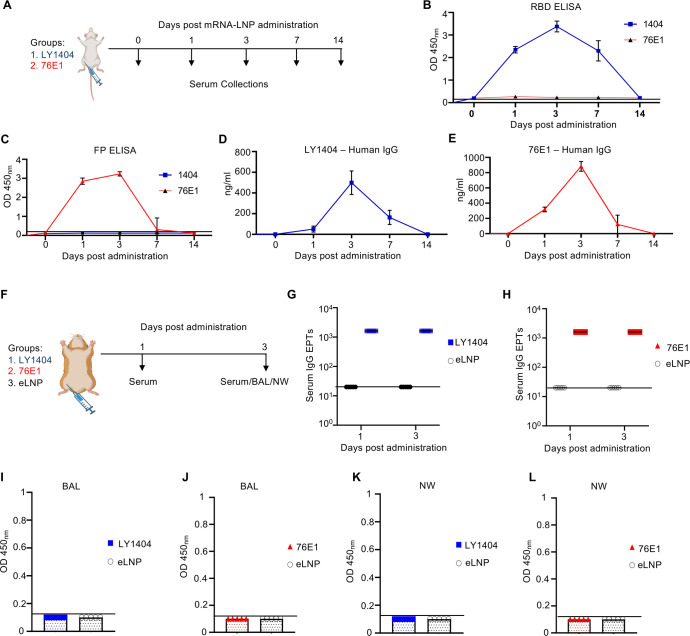
Antibody production *in vivo* in mice and hamsters following mRNA-LNP administration. (**A**) Mouse experimental design and timeline. Two groups of BALB/c mice (*n* = 5/group) were administered IM with mRNA-LNP, followed by serum collection at indicated days for antibody measurement. (**B–C**) Measurement of LY1404 (**B**) and 76E1 (**C**) in sera by binding ELISA. (**D–E**) Measurement of LY1404 (**D**) and 76E1 (**E**) in sera by quantitative human IgG ELISA. (**F**) Hamster experimental design and timeline. Three groups of hamsters (*n* = 5/group) were administered IM with mRNA-LNP, followed by serum collection at 1 and 3 days after administration. At 3 days after administration, hamsters were subjected to terminal harvest. Bronchoalveolar lavage (BAL) fluid and nasal wash (NW) were also collected, in addition to sera. (**G–L**) Measurement of antibody levels in hamster sera (**G–H**), BAL (**I–J**), and NW (**K–L**). LY1404 was measured by RBD-binding ELISA (**G, I, K**) and 76E1 measured by FP-binding ELISA (**H, J, L**). Antibody end point titers (EPTs) in sera were calculated (**G–H**). Antibody levels in BAL and NW were shown as OD450 nm at undiluted samples (**I–L**). All ELISA assays were performed with duplicates, and the data show mean and SEM of five animals in each group. Limit of detection (LOD) for binding ELISA (OD450 nm < 0.1) and for quantitative ELISA (1.56 ng/mL IgG).

Next, we tested if mRNA-LNPs can mediate *in vivo* antibody production in animals. First, two groups of BALB/C mice (*n* = 5/group) were IM administered with a mixture of HC and LC mRNA-LNPs at a 1:1 molar ratio (15 µg total mRNA) encoding either LY1404 or 76E1 (Fig. 2A). Antibody levels in sera were measured at 1, 3, 7, and 14 days post mRNA-LNP administration using antigen-binding ELISA (Fig. 2B and C) or quantitative human IgG ELISA (Fig. 2D and E). RBD-binding ELISA revealed that mice receiving LY1404-encoding mRNA-LNPs produced RBD-reactive antibody in sera as early as day 1, which peaked at day three and remained at high levels through day 7; in contrast, no RBD-reactive antibody was detected in sera from the 76E1 mRNA-LNP group (Fig. 2B). Conversely, FP-binding ELISA showed that mice receiving 76E1, but not LY1404, mRNA-LNPs produced FP-reactive antibodies in sera (Fig. 2C). While LY1404 antibody levels remained high through day 7, 76E1 antibodies declined by this time point (Fig. 2B and C). Human IgG ELISA revealed consistent results, showing robust human IgG expression following IM administration of LY1404- or 76E1-encoding mRNA-LNPs, with kinetics similar to those observed in antigen-binding ELISAs (Fig. 2D and E).

To assess antibody levels at the respiratory mucosa, Syrian golden hamsters (*n* = 5 per group) were IM administered with either empty LNPs (eLNP; negative control) or mRNA-LNPs encoding LY1404 or 76E1 at a 1:1 molar ratio (15 µg total mRNA) (Fig. 2F). BAL fluid and NW samples were collected at day 3 post-administration. Sera were collected at days 1 and 3. Antibody levels in all samples were determined by binding ELISA (Fig. 2F). Measurement of antibody EPTs revealed that mRNA-LNPs encoding either LY1404 or 76E1 resulted in robust antibody production in hamster sera, with EPTs exceeding 10³ at both day 1 and day 3 (Fig. 2G and H; Fig. S4). These results are consistent with the findings observed in mice. However, no or minimal antibodies were detected in BAL and NW of hamsters (Fig. 2I through L).

### Effects of mRNA-delivered antibodies on SARS-CoV-2 control in mice

After confirming *in vivo* expression of LY1404 and 76E1 antibodies following mRNA-LNP administration, we next evaluated their protective effects on SARS-CoV-2 infection in animal models. The initial study was performed utilizing wild-type (WT) mice infected with the MA SARS-CoV-2 CMA4 strain (36). This strain replicates efficiently in mice but exhibits low pathogenicity without causing overt disease or tissue pathology in mice, providing a simple yet rapid model to assess *in vivo* viral suppression mediated by the mRNA-delivered antibodies.

**Fig 3:**
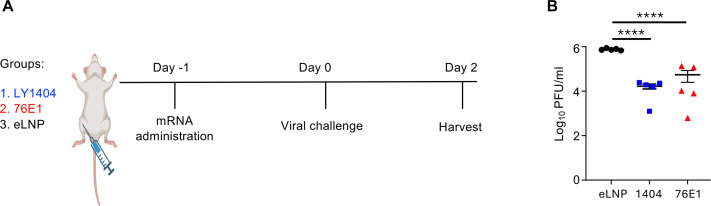
Protective effect of mRNA-delivered antibodies on MA SARS-CoV-2 infection in mice. (**A**) Experimental design and timeline. Three groups of BALB/c mice (*n* = 5/group) were administered IM with antibody-encoding mRNA-LNP (LY1404 or 76E1) or with eLNP as a control. One day after administration, all mice were infected with MA SARS-CoV-2 (CMA4). Two days after viral challenge, animals were terminally harvested for quantification of viral titers in lungs. (**B**) Comparison of lung viral titers among the three groups. ****P < 0.0001.

Three groups of 6-week-old female BALB/C mice were IM administered with eLNP or mRNA-LNPs encoding LY1404 or 76E1 antibodies (HC/LC 1:1 molar ratio; 15 µg total mRNA per mouse) (Fig. 3A). One day post-administration, mice were intranasally infected with MA-SARS-CoV-2 CMA4 (2 × 10^4^ pfu). Two days after infection, mice were euthanized, and viral titers in the lungs were quantified (Fig. 3A). Compared with the eLNP control group, mice receiving LY1404 or 76E1 mRNA-LNPs showed a significant reduction in lung viral titers (Fig. 3B). Specifically, LY1404 mRNA-LNP treatment significantly reduced viral titers by 65-fold, and 76E1 mRNA-LNP treatment led to a significant reduction by 49-fold, relative to eLNP (Fig. 3B). These results demonstrate that mRNA-delivered neutralizing antibodies suppress MA SARS-CoV-2 *in vivo*.

### Protection of mRNA-delivered antibodies against SARS-CoV-2 Omicron BQ.1 in hamsters

**Fig 4:**
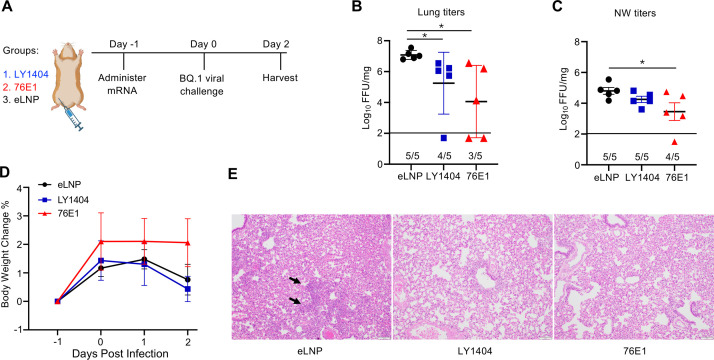
Protection of antibody-encoding mRNA-LNPs against SARS-CoV-2 Omicron BQ.1 infection in hamsters. (**A**) Hamster experimental design and timeline. Three groups of hamsters (*n* = 5/group) were administered IM with antibody-encoding mRNA-LNP (LY1404 or 76E1) or eLNP as a control. One day after administration, all hamsters were infected with SARS-CoV-2 Omicron BQ.1. Two days after viral infection, animals were terminally harvested to determine lung/NW viral titers and lung histopathology. (**B and C**) Comparison of viral titers in the lungs (**B**) and NW (**C**) among the three groups. The number of hamsters with detectable viral titers in each group (*n* = 5) is shown. (**D**) Comparison of hamster body weight changes. Data were shown as % body weight changes to the baseline (Day −1). (**E**) Hamster lung histopathology analysis. Representative lung hematoxylin and eosin (H&E) image for each group is shown. *P < 0.05.

We next evaluated the protective effects of the two mRNA-delivered antibodies against a more immune-evasive SARS-CoV-2 variant. Hamsters are susceptible to infection by both ancestral and variant lineages of SARS-CoV-2, making them a relevant model for evaluating therapeutic agents (37, 38). Three groups of 6-week-old hamsters (*n* = 5/group) were IM administered with either eLNP or mRNA-LNPs encoding LY1404 or 76E1 antibodies (HC/LC 1:1 molar ratio; 15 µg total mRNA per hamster) (Fig. 4A). One day post-administration, hamsters were intranasally infected with the SARS-CoV-2 Omicron BQ.1 variant (2 × 10^4^ PFU), which exhibits marked escape from many neutralizing antibodies (39). Two days after infection, viral titers in the lungs and NW were quantified to assess the impact of mRNA-delivered antibodies on viral replication. In addition, hamster body weights were monitored, and lung histopathology was analyzed to evaluate disease protection conferred by mRNA-delivered antibodies against SARS-CoV-2 BQ.1 (Fig. 4A).

Both LY1404- and 76E1-encoding mRNA-LNPs significantly reduced viral titers in the lungs (Fig. 4B). In the eLNP control group, all five hamsters exhibited high viral titers. In contrast, LY1404-encoding mRNA-LNPs resulted in complete viral suppression in one hamster and significantly reduced titers in the remaining hamsters (Fig. 4B). Similarly, 76E1-encoding mRNA-LNPs led to robust suppression of BQ.1 replication in the lungs, achieving complete viral suppression in two hamsters and reduced titers in the other three hamsters (*P* < 0.05) (Fig. 4B). In the NW, 76E1 mRNA-LNP administration also resulted in a significant reduction in viral titers (Fig. 4C).

Body weight monitoring revealed that young hamsters in the eLNP group maintained steady weight gain even after Omicron BQ.1 infection (Fig. 4D), consistent with previous findings that Omicron variants exhibit reduced pathogenicity in this model (34). A modest trend toward increased weight gain was observed in the 76E1 mRNA-LNP group compared with eLNP controls, although statistical significance was not reached (Fig. 4D).

Lung histopathological analysis further demonstrated protective effects of mRNA-delivered antibodies. SARS-CoV-2 BQ.1 infection caused mild lesions and pathology in the lungs of a few hamsters in the eLNP control group. A representative image is shown (Fig. 4E; left panel). In contrast, all hamsters in the LY1404 or 76E1 mRNA-LNP group exhibited normal bronchial, bronchiolar, and alveolar architecture (Fig. 4E; middle & right panels). Together, these findings support that IM administration of antibody-encoding mRNA-LNPs confers protection against SARS-CoV-2 Omicron BQ.1 infection in hamsters.

### Protection of mRNA-delivered antibodies against SARS-CoV-2 Delta infection in hamsters

**Fig 5:**
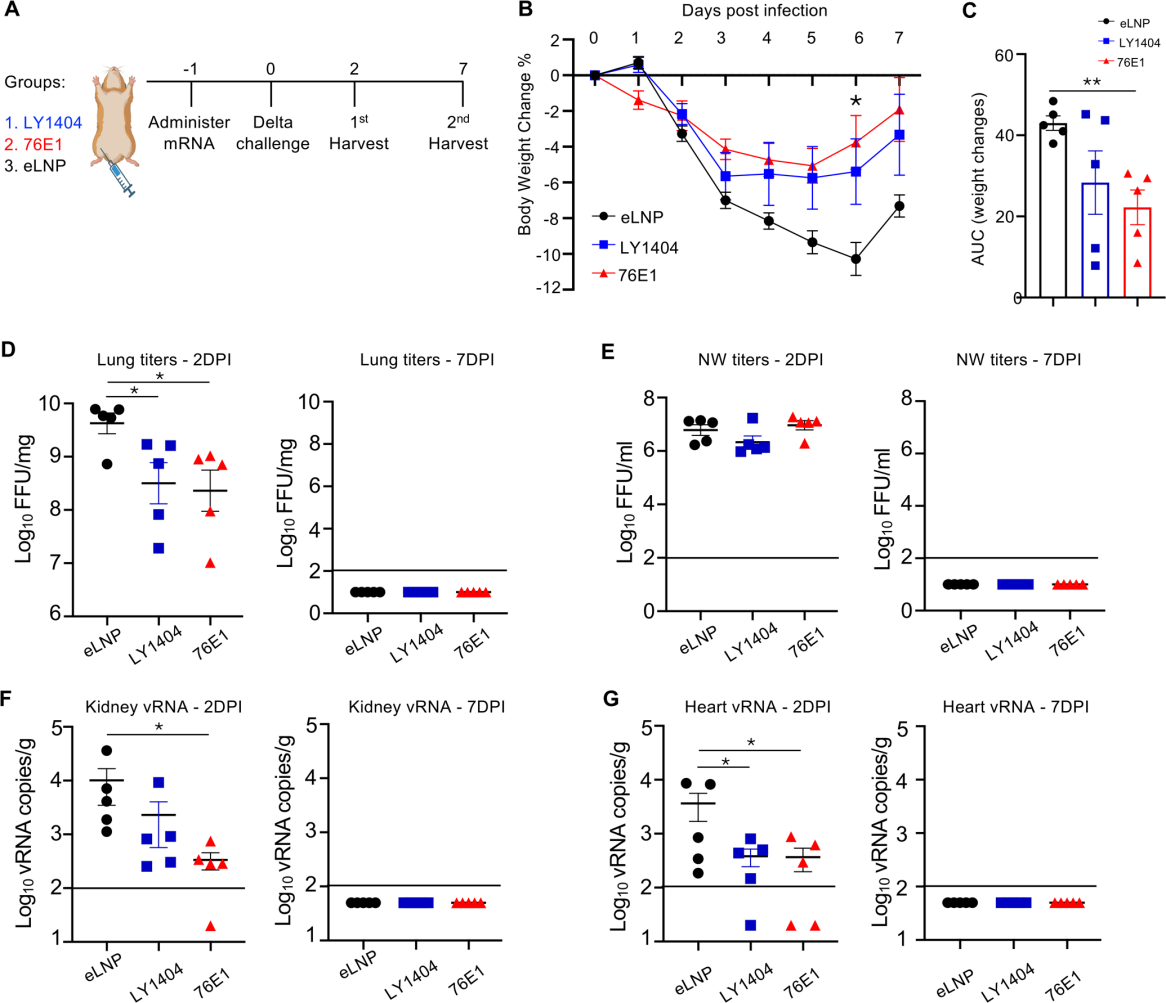
Protection of antibody-encoding mRNA-LNPs against SARS-CoV-2 Delta infection in hamsters. (**A**) Hamster experimental design and timeline. Three groups of hamsters (*n* = 10/group) were IM administered with mRNA-LNPs encoding LY1404 or 76E1 or eLNP. One day after administration, hamsters were infected with SARS-CoV-2 Delta. For each group, half of the cohort (*n* = 5) were terminally harvested at 2 DPI to determine viral loads and the other half (*n* = 5) terminally harvested at 7 DPI to determine disease and pathology. (**B**) Hamster body weight changes from Day −1 to 7 DPI. % body weight changes relative to the baseline (Day −1) are shown. (**C**) Area under the curve (AUC) for weight changes was calculated and compared among the three groups. (**D and E**) Viral titers in the lungs (**D**) and NW (**E**) at 2 and 7 DPI. (**F and G**) Viral RNA (vRNA) copies in the kidney (**F**) and heart (**G**) at 2 and 7 DPI. LOD for each assay is denoted. *P < 0.05; **P < 0.01.

While the MA-SARS-CoV-2 and Omicron BQ.1 infection experiments provided key evidence for *in vivo* antiviral activity of mRNA-delivered antibodies, their protective effects against SARS-CoV-2-induced disease and morbidity have not yet been quantitatively assessed due to the low-to-moderate pathogenicity of these strains in animals. To more rigorously evaluate the protective efficacy of mRNA-delivered antibodies against diseases and morbidity beyond viral replication, we conducted a hamster challenge study utilizing the highly pathogenic SARS-CoV-2 Delta variant. As illustrated in Fig. 5A, three groups of hamsters (*n* = 10 per group) were IM administered with either eLNP or mRNA-LNPs encoding LY1404 or 76E1 antibodies (Day −1), followed by intranasal infection with SARS-CoV-2 Delta (2 × 10⁴ pfu) on Day 0. For each group, five animals were euthanized at 2 days post-infection (DPI) (first harvest) to assess viral loads and the remaining five at 7 DPI (second harvest) to evaluate disease and pathology. Viral loads in lung, NW, kidney, heart, and brain were quantified at both time points. Body weight and lung histopathology were also monitored to assess disease severity (Fig. 5A).

Delta infection caused marked body weight loss exceeding 10% by 6 DPI (Fig. 5B), confirming its high pathogenicity compared to Omicron BQ.1 infection (Fig. 4). Both mRNA-delivered antibodies significantly mitigated weight loss relative to eLNP, with 76E1 showing the greatest effect as determined by AUC analysis (Fig. 5C). At 2 DPI, high viral titers were detected in the lungs and NW (Fig. 5D and E), approximately 2 log₁₀ higher than those caused by Omicron BQ.1 infection (Fig. 4C). Titers declined to undetectable levels by 7 DPI. Both mRNA-delivered LY1404 and 76E1 significantly reduced lung viral titers at 2 DPI (Fig. 5D), although no reduction was observed in NW (Fig. 5E). No infectious virus was detected in the kidney, heart, or brain by the FRNT assay (Fig. S5). However, using more-sensitive RT-qPCR, the analysis revealed low but detectable vRNA in these extrapulmonary tissues (Fig. 5F and G; Fig. S6). Both antibodies reduced vRNA levels, with significant reductions observed for 76E1 in the kidney and heart (*P* < 0.05) and for LY1404 in the heart (*P* < 0.05). A trend toward vRNA reduction was also observed by 76E1 in the brain (Fig. S6).

**Fig 6:**
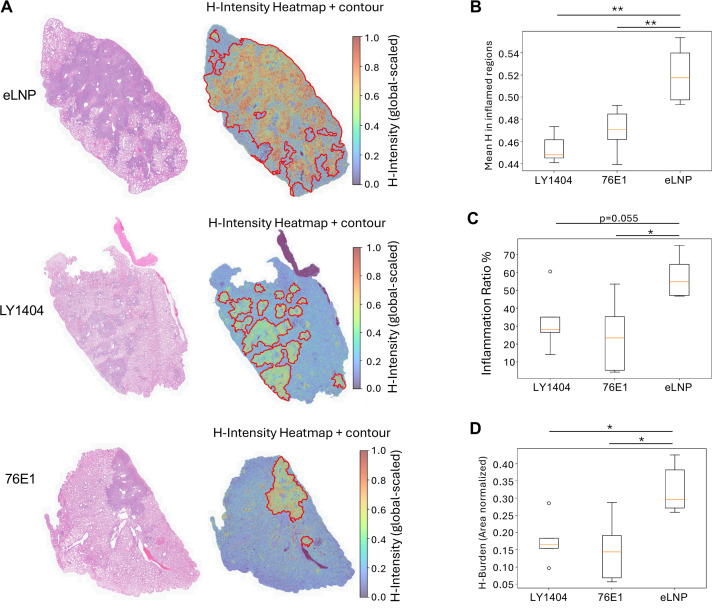
Histopathological analysis of lungs from Delta-infected hamsters. Lung tissues collected at 7 DPI were fixed and stained with H&E for histopathological evaluation. Quantitative image analysis was performed using a custom Python pipeline. (**A**) Representative images of H&E-stained lung sections (left) and corresponding heatmap/contour analyses (right) from each group illustrating the area and extent of inflammation based on hematoxylin (H)-intensity. In heatmaps, globally scaled H-intensity reflects inflammation severity, and contours delineate inflamed tissue regions. (**B**) Cumulative quantification of mean H-intensity within inflamed regions, indicating the severity of inflammation. (**C**) Cumulative quantification of the area-normalized fraction of inflamed tissue (Inflammation Ratio, %), representing the proportion of the inflamed area. (**D**) Cumulative quantification of area-normalized H burden, integrating both the extent and intensity of inflammation. *P < 0.05; **P < 0.01.

Histopathological examination at 7 DPI showed severe lung inflammation and pathology for hamsters in the eLNP group (Fig. 6A; Fig. S7), consistent with their pronounced weight loss (Fig. 5B and C). In contrast, hamsters treated with antibody-encoding mRNA-LNPs displayed markedly attenuated pulmonary pathology, characterized by only mild-to-moderate inflammation. Quantitative image analysis of H&E-stained sections using a custom Python pipeline (Fig. 6A) revealed significant reductions in mean H-intensity, inflammation ratio, and hematoxylin burden for 76E1-treated animals compared to eLNP (*P* < 0.05) (Fig. 6B through D; Fig. S7).

Together, these results demonstrate that mRNA-delivered neutralizing antibodies, particularly 76E1, confer significant protection against SARS-CoV-2 Delta infection in hamsters, reducing both viral burden and disease severity.

## DISCUSSION

The mRNA-LNP platform has attracted considerable attention for its capacity to deliver therapeutic proteins and antibodies as countermeasures against diverse diseases (21–24), in addition to its milestone success in vaccine development. To combat viral infections such as COVID-19, a number of neutralizing antibodies with therapeutic potential have been identified (12). However, large-scale implementation of antibody therapeutics remains limited by the high cost and complexity of antibody production and manufacturing (27, 28). Here, we demonstrate that mRNA-LNPs can effectively and rapidly express two distinct SARS-CoV-2-neutralizing antibodies, LY1404 and 76E1, *in vivo*. A single IM dose of mRNA-LNP (15 µg total mRNA) conferred significant protection against multiple SARS-CoV-2 variants in two animal models. These findings support the potential of mRNA-based antibody delivery as a promising alternative or complement to vaccination, offering rapid protection for high-risk groups such as travelers to endemic regions and immune-compromised individuals.

LY1404 and 76E1 are two SARS-CoV-2-neutralizing antibodies that target distinct regions of the viral S protein (20, 40). Our study demonstrates that both antibodies, when delivered via mRNA-LNPs, significantly control replication of multiple SARS-CoV-2 strains in lungs, including Delta and Omicron BQ.1. Notably, the observed *in vivo* protection conferred by LY1404 against BQ.1 is intriguing as this antibody exhibits potent neutralizing activity against early SARS-CoV-2 strains and Omicron subvariants but shows markedly reduced or lost neutralizing potency against more recent variants (41, 42). In our study, mRNA-delivered LY1404 still induced significant, albeit moderate, reduction of BQ.1 in hamsters, likely reflecting contributions from Fc-mediated effector functions of this antibody. Indeed, a recent study reported that LY1404 possesses moderate antibody-dependent cellular cytotoxicity (ADCC) activity (43). Furthermore, although LY1404 completely lost neutralizing activity against BQ.1.1, it retained a substantially reduced yet detectable IC50 against BQ.1 in certain *in vitro* neutralization assays (44), which may also contribute to the moderate protection observed *in vivo*. Nevertheless, the mechanisms by which mRNA-delivered antibodies protect against antigenically evolved viral variants warrant further investigation in future studies.

IM administration of mRNA-LNPs resulted in robust antibody production in circulation; however, minimal or no antibodies were detected in BAL and NW. This is likely attributable to the fact that both LY1404 and 76E1 antibodies constructed in this study are human immunoglobulin G (IgG), and monomeric IgG exhibits limited transcytosis to mucosal sites due to the absence of an active transport mechanism such as the polymeric Ig receptor (pIgR) used for IgA (45). Consistent with this, IM administration of antibody-encoding mRNA–LNPs did not significantly reduce viral loads in the NW of Delta-infected hamsters, likely reflecting the lack of detectable antibodies at mucosal surfaces. In contrast to IgG, dimeric IgA can be actively transported across epithelial barriers via pIgR and plays a key role in mucosal defense against viral infections (45–47). Future studies are warranted to explore the potential of mRNA-based delivery of secretory or dimeric IgA as a strategy for enhancing mucosal protection against viral infections.

Efficient LNP-mediated delivery of mRNA payloads is fundamental to the success of mRNA vaccines and therapeutics (48–50). In this study, we employed a conventional liver-targeted LNP formulation (SM-102) that has been optimized for IM administration (51, 52), resulting primarily in systemic antibody production. Increasing efforts are being directed toward developing novel LNPs capable of organ- or tissue-specific mRNA delivery (53). Recent advances in ionizable lipids and LNP formulations have enabled enhanced mRNA delivery to the lung (29, 52), and emerging studies have identified novel lipids suitable for efficient intranasal mRNA delivery (54, 55). Our findings demonstrate the feasibility of *in vivo* antibody expression following IM mRNA–LNP administration. Given the superior neutralizing potency and mucosal breadth of dimeric IgA compared to monomeric IgG, together with ongoing progress in site-specific LNP design, future studies should aim to combine these advances to develop mucosal delivery of IgA-encoding mRNAs for robust respiratory protection and prevention of viral transmission.

Our study examined the kinetics of circulating antibodies in mice following a single dose of mRNA-LNP and revealed that both LY1404 and 76E1 were rapidly produced in sera within 24 h. The serum antibodies peaked at 3 days and gradually diminished over time to undetectable levels by 14 days post-mRNA administration. This serum antibody kinetics is consistent with a previous report using similar mRNA-LNP technology to deliver VRCO1, an HIV-1-neutralizing antibody (33). The modest durability of antibody production in our animal models could be attributed to multiple factors, including the transient expression nature of mRNA and immune suppression of human IgG antibodies expressed in the mouse host. One method to prolong antibody expression is repeated mRNA-LNP administration (33). Alternatively, compared to mRNA, other RNA technologies such as circular RNA (56–58) and self-amplifying RNA (59, 60) may provide advantages in delivering therapeutic proteins or antibodies regarding RNA stability and duration of protein expression. These technologies should be explored in the future for expressing therapeutic proteins or antibodies to enhance the duration of protein expression as compared to the linear mRNA platform.

Limitations of the current study are acknowledged. First, it would be ideal to include the protein format of the two antibodies (LY1404 and 76E1) as controls in the animal experiments to more accurately assess the dose-sparing effect of mRNA delivery. The clinical dose of LY1404 for COVID-19 treatment in humans was 175 mg administered as a single intravenous infusion, while other monoclonal antibodies such as tixagevimab, cilgavimab, and sotrovimab have been used at even higher doses (500–600 mg). Based on the human dosing of LY1404, the estimated equivalent dose in hamsters would be approximately 300 µg per animal, which remains substantially higher than the mRNA dose used in this study(33). To more rigorously evaluate the dose efficiency of mRNA-mediated antibody expression, future studies should include direct comparisons between mRNA-encoded and protein antibodies in terms of dosing, pharmacokinetics, and protective efficacy in relevant animal models. Second, this study focused on the prophylactic model in which mRNA-encoded antibodies were administered prior to viral challenge. The therapeutic efficacy of this approach following established infection, as well as the duration of protection, remains to be determined.

In summary, this study provides a proof of concept that administration of mRNA–LNPs encoding human neutralizing antibodies can confer protection against multiple SARS-CoV-2 strains in different animal models. These findings support the potential of mRNA-based antibody delivery as an alternative or complementary approach to vaccination for achieving rapid protection, particularly in high-risk or immunocompromised populations.

## MATERIALS AND METHODS

### Viruses

The Delta and Omicron BQ.1 variants were obtained from the World Reference Center for Emerging Viruses and Arboviruses at UTMB. Briefly, viral isolates were derived from RT-qPCR-confirmed positive human samples, followed by titration and propagation in Vero TMPRSS2 cells. Virus stocks were harvested at approximately 30% cytopathic effect, aliquoted, and deep-sequenced using an Illumina platform. Only sequence-confirmed stocks were used for *in vivo* studies. Generation of the MA SARS-CoV-2 CMA4 strain has been described previously (36). In brief, the earlier CMA3 strain was serially passaged in mice for 20 passages to promote adaptation, and the CMA4 strain was subsequently engineered by introducing identified adaptive mutations into the CMA3 backbone (36).

### mRNA synthesis and purification

mRNA synthesis and purification were conducted following previously reported protocols with minor modifications (34). Briefly, the HC and LC gene sequences of LY1404 and 76E1 were codon-optimized, synthesized, and cloned into DNA vectors. Nucleoside-modified mRNAs were transcribed *in vitro* from linearized DNA templates using the HiScribe CleanCap mRNA IVT kit (NEB), with uridine substituted by N1-methyl-pseudouridine. The resulting mRNAs were precipitated from the IVT reaction using lithium chloride and subsequently purified by cellulose-based chromatography to remove residual double-stranded RNA (35). Purified mRNAs were analyzed by gel electrophoresis and Agilent bioanalyzer to assess integrity, quantified for concentration, and stored at −20°C for further characterization and LNP formulation.

### Measurement of LY1404 and 76E1 antibody production by cells

Approximately 2 × 10⁵ 293T cells were seeded in 12-well plates 1 day prior to mRNA transfection. HC and LC mRNAs for either LY1404 or 76E1 were co-transfected at a 1:1 molar ratio using a total of 2 μg mRNA per well with the Lipofectamine MessengerMAX transfection reagent (Thermo Fisher Scientific). Culture supernatants were collected 24 h post-transfection for assessment of antibody expression by ELISA and WB. ELISA was performed using either the corresponding antigen or a human IgG capture antibody, following previously described procedures (34). WB analysis was conducted to confirm the expression of fully assembled antibodies. Briefly, collected supernatants were mixed with Laemmli buffer at a 1:1 volume ratio and boiled at 95°C for 10 min. LY1404 and 76E1 IgG proteins were detected using an anti-human IgG lambda LC antibody (1:3,000; Abcam). Membranes were incubated with the primary antibody in washing buffer containing 5% milk overnight at 4°C, followed by incubation with a horseradish peroxidase (HRP)-conjugated secondary antibody (1:3,000) for 1 h at room temperature. After washing, the blots were visualized using an enhanced chemiluminescence substrate (Thermo Fisher Scientific).

### mRNA-LNP encapsulation

mRNAs were diluted in citrate buffer (20 mM, pH 4) to a final concentration of 0.2 µg/µL to serve as the aqueous phase. The lipid organic phase, composed of ionizable lipid (SM-102), DSPC, cholesterol, and DMG-PEG-2000, was prepared in ethanol at a molar ratio of 50:10:38.5:1.5 (individual lipid vendors). The aqueous and organic phases were combined using a NanoAssemblr microfluidic system (Cytiva) at a 3:1 flow-rate ratio and a total flow rate of 12 mL/min. The resulting mRNA–LNPs were dialyzed against 30 volumes of Dulbecco’s phosphate-buffered saline (dPBS), concentrated using Amicon Ultra centrifugal filters (10 kDa MWCO), and sterilized through 0.22 µm Pall syringe filters. Particle size and PDI were determined by dynamic light scattering (DLSusing a Malvern Zetasizer）). The mRNA concentration and encapsulation efficiency of the formulated LNPs were quantified using the Quant-iT RiboGreen RNA assay kit (Thermo Fisher Scientific). Following characterization, sterile-filtered mRNA-LNPs were stored at −80°C until further use.

### Antigen-binding ELISA

Antigen-binding ELISAs were performed to assess the expression, kinetics, and antigen specificity of antibodies in experimental samples, following a previously described protocol with modifications (34).

For Spike and RBD ELISA, plates were coated overnight at 4°C with dPBS containing 1 µg/mL recombinant SARS-CoV-2 S or RBD protein (Sino Biological). On the following day, plates were washed twice with PBS containing 1.5% Tween-20 and blocked for 1 hour at 37°C with dPBS supplemented with 8% fetal bovine serum (FBS). After two washes, experimental samples such as culture supernatants, animal sera (diluted 1:20 in blocking buffer), or BAL/NW were added and incubated for 1 hour at 37°C. Plates were then washed five times and incubated with horseradish peroxidase (HRP)-conjugated anti-human IgG antibody (Abcam) at a 1:3,000 dilution in wash buffer for 1 hour at 37°C. Following a final wash, plates were developed using the TMB 1-Component Peroxidase Substrate (Thermo Fisher Scientific), and the reaction was stopped with TMB Stop Solution (Thermo Fisher Scientific). Absorbance was measured at 450 nm within 15 minutes using a BioTek Microplate Reader. Limit of detection (LOD) for the assay was defined as an OD₄₅₀ value of 0.1.

For FP ELISA, biotinylated 15-mer overlapping peptides spanning amino acids 816–835 of SARS-CoV-2 S protein were synthesized (JPT Peptide Technologies) and reconstituted in peptide coating buffer at 1 mM. The peptides were incubated on streptavidin-coated plates (Invitrogen) for 1 hour at room temperature, followed by five washes and blocking with dPBS containing 10 mg/mL bovine serum albumin (BSA) for 1 hour at room temperature. Subsequent incubation and detection steps were performed as described above in the SpikeS and RBD ELISAs.

### Human IgG ELISA

Human IgG ELISA with standards was performed to quantify antibody concentrations in experimental samples. ELISA plates were coated overnight at 4°C with capture anti-human IgG antibody diluted 1:1,000 in dPBS. The next day, plates were washed six times and blocked with dPBS containing 5% milk for 1 hour at 37°C. After blocking, plates were allowed to dry without additional washing. Samples diluted 1:20 in dPBS with 2% milk were added and incubated for 1 hour at 37°C, followed by six washes. HRP-conjugated anti-human IgG secondary antibody (1:3,000) was then applied for 1 hour at 37°C. After final washes, plates were developed with the TMB substrate, and absorbance was measured at 450 nm using a microplate reader, as described above. Antibody concentrations were calculated based on a standard curve generated from human IgG standards. LOD for the assay was 1.56 ng/mL.

### Mouse infection with MA SARS-CoV-2 CMA4 and quantification of lung viral titers

Six-week-old female BALB/c mice were IM injected with mRNA-LNPs encoding the HC and LC of LY1404 or 76E1, or with eLNP as a negative control. The HC and LC mRNA-LNPs were administered at a 1:1 molar ratio, with a total mRNA dose of 15 µg per mouse. Twenty-four hours post-mRNA administration, all mice were intranasally infected with 2 × 10⁴ PFU of the MA SARS-CoV-2 CMA4 strain. At 2 days post-infection, all mice were euthanized, and lung tissues were collected for viral titer quantification by plaque assay, as previously reported (61). In brief, Vero E6 cells were seeded in 6-well plates and incubated at 37°C. Lung tissue homogenates were serially diluted 10-fold in Dulbecco’s modified Eagle’s medium containing 2% FBS, and 0.2 mL of each dilution was used to infect the cells for 1 hour at 37°C. After infection, the inoculum was removed, and cells were overlaid with 2× MEM (Gibco) supplemented with 8% FBS and 1.6% agarose (Promega). Following a 48-hour incubation, plates were stained with 0.05% neutral red (Sigma-Aldrich), and plaques were enumerated to determine viral titers.

### Hamster infection with SARS-CoV-2 Omicron BQ.1 or SARS-CoV-2 Delta

Six-week-old male Syrian golden hamsters (HsdHan; Inotiv) were I.M administered mRNA-LNPs encoding LY1404 or 76E1, or with eLNP as a negative control. The HC and LC mRNA-LNPs were mixed at a 1:1 molar ratio for administration, with a total mRNA dose of 15 µg per hamster. Twenty-four hours post-mRNA administration, hamsters were intranasally infected with SARS-CoV-2 Omicron BQ.1 or Delta variant (2 × 10⁴ PFU). In the BQ.1 infection experiment, all animals were euthanized at 2 DPI for quantification of viral titers in the lungs and NW as well as for lung histopathological analysis. In the Delta infection experiment, hamsters were euthanized at 2 and 7 DPI for terminal sample collection. Lung, NW, kidney, heart, and brain tissues were collected for viral load quantification, and lung tissues were examined for histopathological changes.

### Quantification of SARS-CoV-2 viral titers in hamster

Viral titers were quantified as previously described (34). Viral titers were quantified as previously described (34). Briefly, homogenized tissues or NW samples were serially diluted up to 10⁶ in maintenance medium and added to confluent Vero E6 cell monolayers (ATCC CRL-1586) in 96-well plates for 1 hour at 37°C. After incubation, an overlay medium was added, and plates were incubated for 48 hours. Cells were then fixed with 10% formalin, washed with dPBS, and permeabilized with dPBS containing 0.1% BSA and 0.1% saponin for 30 minutes at room temperature. Monolayers were incubated overnight at 4°C with rabbit polyclonal anti-SARS-CoV nucleocapsid antibody (1:3,000; S. Makino, UTMB), followed by HRP-conjugated goat anti-rabbit IgG (1:2,000; Cell Signaling Technology) for 1 hour at room temperature. After final washes, foci were visualized using KPL TrueBlue Peroxidase Substrate (SeraCare), and the reaction was stopped. Plates were read on a microplate reader, and foci were counted to determine viral titers.

### Quantification of SARS-CoV-2 viral RNAs in hamsters

One-step RT-qPCR for quantification of viral RNAs in tissues was performed using the iTaq Universal SYBR Green One-Step Kit (Bio-Rad) and CFX Connect Real-Time PCR Detection System (Bio-Rad), as previously described (34). Primer sets targeting SARS-CoV-2 E gene were used (F: 5′-GGAAGAGACAGGTACGTTAATA-3′; R: 5′-AGCAGTACGCACACAATCGAA-3′). RT-qPCR reaction included the following: 10 μM primer, 2 μL RNA template, 10 μL iTaq Universal SYBR Green Supermix, 0.25 μL iScript reverse transcriptase, and molecular-grade water. The amplification protocol: an initial denaturation at 95°C for 3 min, followed by 45 cycles of 95°C for 5 s and 60°C for 30 s. For absolute quantification, a standard curve was generated using an *in vitro*-transcribed RNA standard to determine viral RNA copy numbers (34).

### Lung histopathology

Hamster lungs were fixed in 10% neutral-buffered formalin, embedded in paraffin, sectioned, and stained with hematoxylin and eosin (H&E) following a previously described protocol (34). In the Omicron BQ.1 infection study, lung sections were independently evaluated by a board-certified pathologist. For the Delta infection study, staining slides were independently assessed by the research team at Eureka Biotech Inc. Quantitative image analysis was conducted using a custom Python pipeline (https://www.python.org/) (62). The following metrics were derived to quantify lung inflammation and pathology across the experimental groups: (i) mean hematoxylin (H) intensity within inflamed regions, reflecting the severity of nuclear staining in segmented lesions; (ii) inflammation ratio (%), defined as the area-normalized fraction of inflamed tissue; and (iii) area-normalized H burden, calculated as the sum of H intensity within the inflammation mask, integrating both the extent and intensity of inflammation. All lung histopathology analyses were performed with sample identities blinded to the pathologist or analyst.

### Statistics

Statistical analysis was performed using the GraphPad Prism 10 software. Data are presented as mean with SEM or median with interquartile range as indicated. One-way ANOVA was used for statistical comparisons. *P* values < 0.05 were considered significant.

## Data Availability

All data of this study are provided within the paper and its supplemental material.

## SUPPLEMENTAL MATERIAL

Supplemental figures jvi.01897-25-s0001.docx Figures S1 to S7.

ASM does not own the copyrights to Supplemental Material that may be linked to, or accessed through, an article. The authors have granted ASM a non-exclusive, world-wide license to publish the Supplemental Material files. Please contact the corresponding author directly for reuse.
