# Supplementary material for: mRNA-delivered neutralizing antibodies confer protection against SARS-CoV-2 in animal models
Source: J Virol. 2026 Jan 7;100(2):e01897-25. doi: 10.1128/jvi.01897-25 (PMC12911912; doi:10.1128/jvi.01897-25)
Supplement: Supplemental figures — Figures S1 to S7. [file jvi.01897-25-s0001.docx]

**Supplementary Figures**

**mRNA-delivered neutralizing antibodies confer protection against SARS-CoV-2 in animal models**

Nicholas C. Hazell et al.

**Figure S1: Integrity of LY1404 and 76E1 HC/LC mRNAs.** Following *in vitro* transcription and mRNA purification, the integrity and size of all four mRNAs, including those encoding LY1404 HC and LC (**A**) as well as those encoding 76E1 HC and LC (**B**), were analyzed by an Agilent Bioanalyzer. The graphs show the integrity, based on RNA integrity (RIN) score, and size (nucleotide numbers) of the target mRNAs for LY1404 HC/LC **(A)** and 76E1 HC/LC **(B)**.

**Figure S2. ELISA titration curves for LY1404 and 76E1 antibodies produced by cells after mRNA transfection.** Supernatants collected from 293T cells at various days after mRNA transfection (as described in **Figure 1**) were serially diluted and tested for antibody production using antigen-binding ELISA (**A-D**) or quantitative human IgG ELISA (**E-F**). **(A-D)** Binding ELISA was conducted against SARS-CoV-2 spike (A-B), RBD (C), or fusion peptide (D). Absorbance at 450 nm (OD450nm) was plotted against the supernatant dilutions. **(E-F)** Quantitative ELISA was conducted to measure LY1404 or 76E1 concentrations. IgG antibody concentration (ng/ml) was plotted against the supernatant dilutions. In this figure, each curve represents one time point of supernatant collection after mRNA transfection.

**Figure S3: mRNA-LNP encapsulation and characterization. (A)** Individual lipids used for LNP formulation. **(B-C)** Physiochemical characterization of mRNA-LNP particles. (B) LNP size distribution and polydispersity index (PDI) were measured by a dynamic light scattering (DLS) analyzer. Representative graphs for the four mRNA-LNPs are shown. **(C)** Ribogreen assay to determine mRNA encapsulation efficiency (EE%) in LNPs. The assay was performed using the RNA standards provided in the kit. Standard curve was generated based on the concentrations of serially diluted RNA (ug/ml; y-axis) and the corresponding relative fluorescence unit (RFU) (x-axis). Concentration of mRNA was determined using the standard curve and the mRNA encapsulation efficiency (EE%) in LNPs was calculated. **(D)** Table summarizing the PDI, sizes, and mRNA EE% of the four mRNA-LNPs.

**Figure S4. LY1404 and 76E1 IgG titration curves in hamster serum.** Following mRNA-LNP administration, sera from hamsters (n=5) at both day 1 and day 3 were originally diluted at 1:20 followed by seven 3-fold serial dilutions to measure IgG end point titers (EPTs). For LY1404, sera were measured against RBD **(A-B);** For 76E1, sera were measured against FP **(C-D)**. Each curve represents serum of individual hamsters receiving either LY1404 or 76E1 mRNA-LNPs. Data are shown as OD 450_nm_ plotted against serum dilution factors (supplementary to main Figure 2G-H).

**Figure S5. SARS-CoV-2 viral titers in different tissues of hamsters infected with Delta.** Following mRNA-LNP administration and SARS-CoV-2 Delta infection, hamsters were subjected to terminal harvest on 2 and 7 DPI (n=5 per group at each time point) as described in **Figure 5**. In addition to lungs and NW shown in Figure 6, kidney, brain, and heart tissues were also collected and viral titers were quantified using the same FRNT assay **(A-C).**

**Figure S6. SARS-CoV-2 viral RNA copies in hamster brain.** Following mRNA-LNP administration and SARS-CoV-2 Delta infection, hamsters were subjected to terminal harvest on 2 and 7 DPI as described in **Figure 6**. In addition to the lungs and NW, kidney, brain, and heart tissues were also collected for the quantification of viral loads. The data shows viral RNA copies in the hamster brains quantified by RT-qPCR (Log10 vRNA copies/g).

**Figure S7. Quantitative analysis of hamster lung inflammation and pathology.** Hamster lung tissues collected at 7 DPI following Delta infection were subjected to quantitative histopathological evaluation using a custom Python pipeline (described in **Figure 6**). **(A)** For each H&E-stained lung section, three metrics were generated to quantify lung inflammation and pathology, including globally-scaled H-intensity in inflamed region (H-mean-inflam), area-normalized H burden (H burden_area_norm), and area-normalized fraction of inflamed tissue (Inflammation_ratio_percent). The table shows individual values of each metric for the 5 hamsters in each experimental group (eLNP, LY1404, 76E1). **(B)** Statistical summary of the data for each metrics (median and IQR) among the three groups.
